# A hierarchical view on material formation during pulsed-laser synthesis of nanoparticles in liquid

**DOI:** 10.1038/srep16313

**Published:** 2015-11-09

**Authors:** Shyjumon Ibrahimkutty, Philipp Wagener, Tomy dos Santos Rolo, Dmitry Karpov, Andreas Menzel, Tilo Baumbach, Stephan Barcikowski, Anton Plech

**Affiliations:** 1 ANKA/Institute for Photon Science and Synchrotron Radiation, KIT Karlsruhe, Germany; 2 CENIDE and Dept. of Chemistry, University Duisburg-Essen, Germany; 3 Swiss Light Source, Paul-Scherrer Institute Villigen, Switzerland; 4 Laboratory for Application of Synchrotron Radiation, KIT Karlsruhe, Germany

## Abstract

Pulsed-laser assisted nanoparticle synthesis in liquids (PLAL) is a versatile tool for nanoparticle synthesis. However, fundamental aspects of structure formation during PLAL are presently poorly understood. We analyse the spatio-temporal kinetics during PLAL by means of fast X-ray radiography (XR) and scanning small-angle X-ray scattering (SAXS), which permits us to probe the process on length scales from nanometers to millimeters with microsecond temporal resolution. We find that the global structural evolution, such as the dynamics of the vapor bubble can be correlated to the locus and evolution of silver nanoparticles. The bubble plays an important role in particle formation, as it confines the primary particles and redeposits them to the substrate. Agglomeration takes place for the confined particles in the second bubble. Additionally, upon the collapse of the second bubble a jet of confined material is ejected perpendicularly to the surface. We hypothesize that these kinetics influence the final particle size distribution and determine the quality of the resulting colloids, such as polydispersity and modality through the interplay between particle cloud compression and particle release into the liquid.

Large efforts are devoted to the controlled synthesis of nanoparticles with defined composition, sizes or shapes. The production of nanoparticles by PLAL is limited in throughput and particle control as compared to wet-chemical synthesis methods. On the other hand PLAL is capable of producing particles from wide classes of materials, as the underlying process, the plasma formation at a target irradiated by an intense, pulsed laser is applicable to virtually any material^1^,^2^,^3^. Additionally, particles produced by ablation in a liquid, i. e. water, are free of chemical side products or specific ligands, which could compromise the further application of the nanoparticles.

As a consequence the interest in exploiting PLAL is growing^4^,^5^,^6^,^7^,^8^,^9^. However, there is still a considerable lack of understanding of the most fundamental processes during this procedure. The initial laser-induced heating of the target is followed by plasma ignition and expansion^5^,^7^,^10^,^11^,^12^,^13^. During this early interaction ions, atom clusters as well as particles are probably emitted. In liquid the particle emission is limited by the barrier of the fluid^14^. Shock wave emission and consecutively an explosive boiling of the liquid follow. A bubble is formed and expands for a sub-millisecond time span, until energy is dissipated and the bubble collapses again^15^,^16^. Particle formation has already been observed *in-situ* by X-ray scattering methods within this cavity and has been detected to lesser extent outside the cavity within the first tens of microseconds^17^. The interaction of the bubble with the enclosed particles potentially constitutes the critical step for defining the primary particle size.

In a mechanistic view, target elements are ablated and trapped inside the laser-induced cavitation bubble^18^,^19^. Addressing the very early phase of plume expansion, the Sakka group has recently shown correlated images of plasma and bubble in the first few tens of nanoseconds, both having a similar non-hemispherical cloudy-shaped micrometer scale contour^20^ indicating the plasma boundary being in contact with the liquid in the early expansion phase. The subsequent formation of nanoparticles by nucleation and particle growth inside the expanded cavitation bubble is dominated by mutual interactions of different species during plasma cooling, and a nucleation time of at least a few microseconds^13^,^20^. Small particles with diameter around 10 nm are supposed to form very early within a couple of microseconds after plasma ignition^21^, not only under liquid confinement conditions, but also during ablation in air^22^. Amans group also concluded from their dynamic plasma observations during PLAL that nanoparticle nucleation time after plasma cooling lasts at least a few microseconds^13^. De Giacomo group concluded in their recent review that microsecond-scale bubble shrinking dynamics allow nanoparticles to be formed in quasi-constant thermodynamic conditions, after which the nanoparticle transfer in solution happens in the course of bubble collapse^23^.

These primary particles start to interact, grow further and agglomerate into larger units, the so-called secondary particles. Within the first bubble larger particles 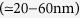 are observed to limited extent on the upper part of the bubble volume. Bubble collapse has been shown to retract a large part of the mass towards the target surface. With compression and remaining heat release from the target a new, smaller bubble forms and drags particle mass away, resulting in a marked bimodal distribution of particles^17^,^24^. A collapse of the second bubble follows with some, yet undefined kinetics afterwards. Results with visible-light shadowgraphy and light scattering have also found indications for directed mass ejection from the target^14^,^21^,^25^, but were not able to resolve the nanoscale structure. In wire target geometry this clearly formed jet is caused by the inertia gained by the collapsing bubble wrapped non-spherically around the wire^14^. The final particle yield, as inspected by electron microscopy retains the bimodal size distribution of primary particles loosely agglomerated to secondary structures as well as a few massive large particles.

In case of ultrashort laser pulses the laser pulse duration is far shorter than the plasma plume lifetime while in the case of nanosecond pulses the plasma is further excited by the laser pulse, believed to be responsible for homogenization of the ejected material^26^,^27^ and hence more narrow (monomodal) size distributions. In the case of femtosecond laser ablation at high laser fluence, photomechanical ablation causes explosive boiling, resulting in bimodal particle size distributions^28^. Peaked bimodal size distribution is not always evident from *post-mortem* analysis, as long-term aggregation or secondary irradiation of pre-existing particles often masks the primary mechanism^29^. With regard to PLAL literature, it has to be noted that mostly number-weighted particle size histograms are reported, naturally underestimating the second mode of larger particles. In particular TEM analysis suffers from bad statistics during counting of these mass-abundant particles often relatively negligible in number. Accordingly, Marzun *et al.* showed during her investigation on size control during picosecond laser ablation of palladium in water that number-weighted size distribution gave a monomodal log-normal size distribution, but mass-weighted statistics clearly revealed a larger fraction with significant intensity, resulting in a bimodal size histogram^30^. Also for nanosecond laser ablation a bimodal distribution of noble metal nanoparticles fabricated by PLAL is revealed for the mass-weighted statistics, even if less pronounced as compared to ultrashort-pulsed, high-fluence ablation. Number-weighted platinum particle size derived by TEM and analytical disc centrifugation (ADC) both show monomodal particle sizes, but mass-weighted statistics clearly show a second mode, and in the TEM also such larger solid spheres are visible^31^. Note that both X-ray extinction (radiography) and scattering (SAXS) signal intensity is proportional to the volume (mass), rather than the number, hence mass-weighted particle size histograms are more appropriate for interpretation of *in situ* experiments done by X-ray probing.

Quality and morphology of recuperated particles are likely to be directly linked to the primary events^22^,^32^. Mastering the interaction of bubble dynamics and particle formation therefore is one important point for controlling particle yield and quality. We have recently seen that the size quenching during PLAL^33^ in the presence of electrolytes is based on surface charge delivery^34^.

The tools for spatially and temporally resolving these delicate processes are continuously evolving, in particular ultrafast imaging methods by light transmission and scattering^25^ and X-ray methods^17^,^35^,^36^. Here we employ two X-ray based techniques, radiography and SAXS, both with microsecond time resolution. Apart from the higher resolving power X-ray beams are less distorted by inhomogeneous morphology of interfaces or opaque phases of the ablation. X-ray radiography is the counterpart to light shadowgraphy being sensitive to light absorption due to the photoelectric effect in dense material. It also contains a phase-sensitive contribution, depending on the propagation distance after crossing the sample. The latter is similar to *Schlieren* photography and based on a deformed wavefront yielding light and dark regions on the detector in materials with inhomogeneous distribution of refractive index. SAXS, in contrast, is sensitive to the nanometer scale. It yields quantitative information both on the absolute mass of the scatterers and on the particle size. It is used for characterizing the size distribution as well as interparticle correlations. Particle aggregation and loose agglomeration, however, cannot be distinguished using SAXS alone. Because of its single-scattering character, it is quantitative as well in terms of determination of absolute mass of scatterers. The signal stems from the entire illuminated area of the sample. Spatial information can thus be gained by employing a pencil beam and raster scanning the sample through the beam. Therefore, combining both radiography and SAXS (Fig. 1) is a powerful tool for spatial imaging on the microscale and quantitative determination of both nanoparticle size and abundance. This correlative approach is needed to reconstruct the kinetics during laser ablation in liquids.

## Results and Discussion

### Bubble kinetics and radiography

The non-equilibrium cavitation bubble kinetics are well described in previous studies experimentally as well as theoretically. The basic motion is that of a size-oscillating bubble out of equilibrium undergoing subsequent expansion and collapse phases. Generally this motion is described by the Rayleigh-Plesset equation^37^. Bubble wall motion follows a parabolic expansion with bubble sizes and lifetime scaling linearly^38^, which has been verified in many different experiments from the nanoscale to millimeter length scales^39^,^40^,^41^,^42^. Important shape modifications of the bubble happen close to a solid boundary. These studies also include the formation of a jet towards the interface^43^, which can cause material abrasion in cavitating mechanical devices. The dynamics of a bubble with origin exactly at the interface, in contrast, can again resemble the free bubble motion^15^,^44^. Shima and Sato^45^, have described the dynamics solving the equation of motion as function of the three-phase boundary angle between vapor, liquid and solid. At an angle of 90° the collapse is as symmetric as the bubble expansion, showing in particular a hemispherical collapse phase. At larger angles (non-wetting case) the bubble would flatten and develop a neck close to the surface, while for smaller angles (water tends to wet the interface) an initial lateral collapse is followed by a jet formation towards the interface, which is known as being responsible for surface damages during cavitation^43^. A sketch of interface motion is depicted in the supporting information, Figure S1. Both cases should distinctly influence the redeposition of laser-ablated material inside the bubble towards the surface, which can significantly reduce the productivity of PLAL as compared to using target shapes that minimize redeposition, such as wires^14^,^25^.

**Figure 2:**
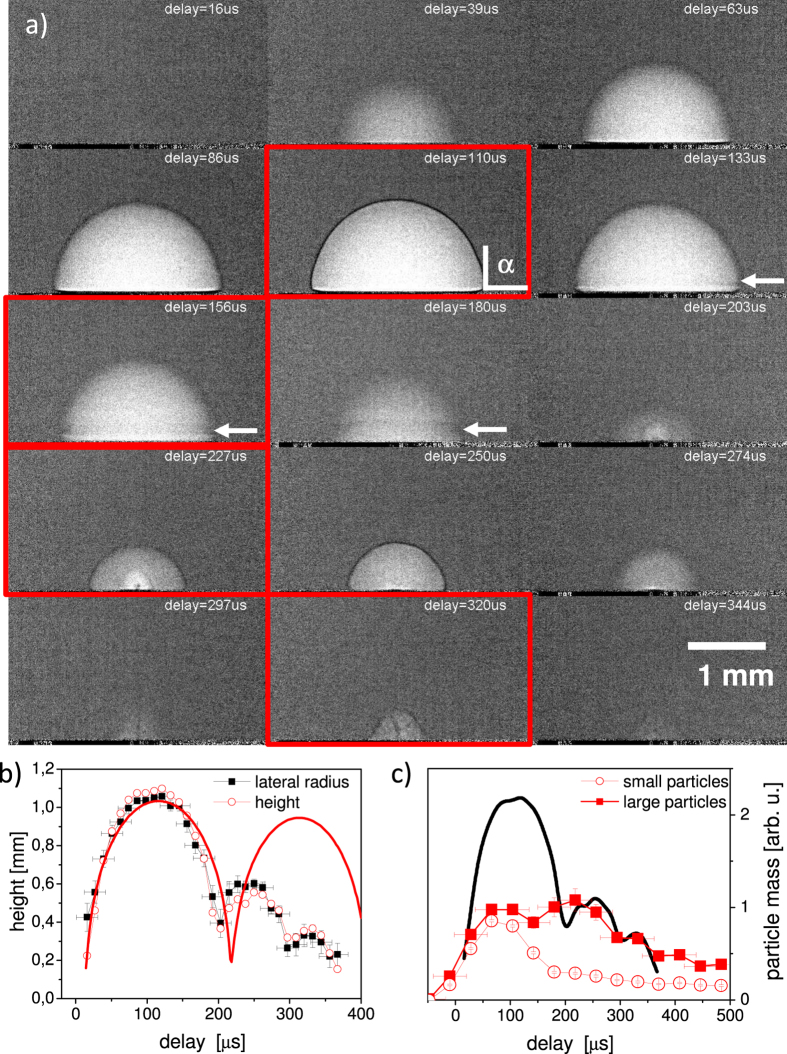
(**a**) Averaged X-ray radiographs of the bubble kinetics at selected delays after laser impact, some critical frames are highlighted: the largest extension of the first bubble at 110 *μs*; shrinking of the first bubble at 156 *μs*; second bubble (rebound) at 227 *μs*; jet formation after the second bubble has collapsed at 320 *μs*. Brighter pixels correspond to higher X-ray transmission. (**b**) plot of the lateral and vertical bubble radius as function of delay. Error bars are in both cases similar. The solid line is a simulation according to the Rayleigh-Plesset equation. (**c**) Mass of ablated particles at a height of 0.5 mm as function of delay. The line indicates the bubble size change according to (**b**). The arrows mark the rim. The contact angle is indicated by *α*.

X-ray radiography films have been taken with an effective frame rate of 85 kHz during the ablation process on a flat silver target. Bubble formation is visible due to the increased X-ray transmission at that location^17^,^24^. Indeed the radiographs in Fig. 2 and supporting material (X-ray radiography film) show a hemispherical transmission increase (bright area) within the forming bubble. Thus the main contrast mechanism is X-ray absorption. There is an additional black rim around the bubble outer surface. This rim is probably caused by differential phase contrast. Such contrast appears at edges of the imaged object with a strong gradient of the refractive index. At the white-beam of a bending magnet, phase contrast is not expected to be pronounced. On the other hand, the distance of the camera from the sample, the beam hardening, and the very high refractive index enhance such propagation phase contrast sufficiently to be observable^46^.

At maximum expansion at 110 *μs* (first highlighted frame in Fig. 2a) the bubble shape is perfectly hemispherical, which is seen by an equal radius in both directions. The bubble appears homogeneous with no indication of strong concentration of ablated mass neither in the center nor close to the phase boundary. However, at this signal-to-noise ratio only areas of dense clustering would be visible. During collapse the shape does not change significantly, while, however, a weak neck forms close to the interface and the height retracts faster than the width (indicated by an arrow in Fig. 2). Figure 2b depicts the derived bubble radius in height and lateral extension. The related flattening is also observed with X-ray transmission recording during scanning SAXS (see supporting information, Figure S1, where also the evolution of the bubble surface is sketched.) This observation infers a large contact angle at the three-phase boundary. This is not necessarily caused by non-wetting conditions of water on the metal surface, but possibly by the fact that the surface is still hot at bubble collapse and maintains a vapor cushion. This had been observed previously^47^ as a change from 90 degrees towards a 120 degree contact angle. Particles confined within the bubble would thus be retracted homogeneously and efficiently towards the target.

**Figure 3:**
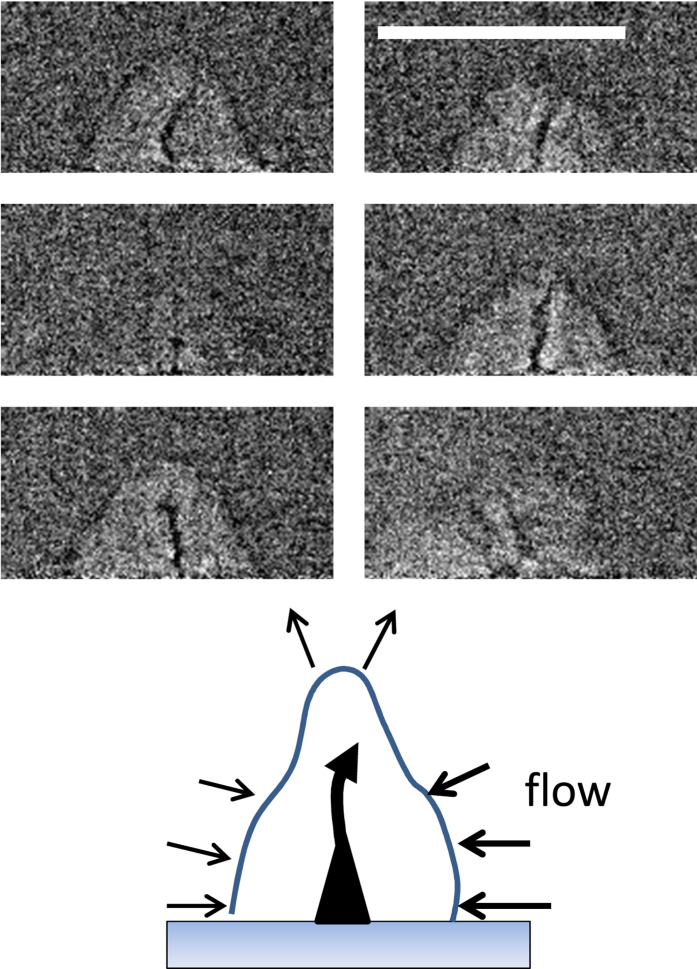
Representative individual radiographs from single laser shots at a fixed delay of ca. 320 *μs* showing the variability of the material ejection. A sketch of the presumptive flow is shown below. The scale bar is 1 mm.

The observation of the collapse event is smeared out due to the limited time resolution of the singularity in wall motion. The smallest quantifiable radius is 0.3 mm as compared to the maximum bubble radius of 1.1 mm. The frame at collapse is completely smeared out. A second bubble (rebound) peaks at 260 *μs* and contains structured contrast features, the nature of which is not easily identified. While the first bubble is well described by the general bubble dynamics in the Rayleigh-Plesset picture (bold line in Fig. 2(b)), the second bubble deviates from this behaviour. The internal structure at 227 *μs* with areas of different densities could mark the interaction of the collapsing bubble with continued boiling at the hot target surface. In fact, at the collapse point the size of the remaining cavity approached the size of the laser spot on the target. Therefore, a singular collapse and subsequent expansion as in sonoluminescence cannot be expected. Internal structure has been seen previously^19^,^48^. In particular in CO_2_ matrix close to the critical point the continuous heat supply from the target creates additional voids and dephasing of the bubble dynamics. After collapse of the second bubble yet another structure evolves, which shows a different shape. Besides a cap-like envelope a thin dark structure within the lighter bubble appears. The frames in Fig. 2(a) are, in fact, an average over 24 individual shots, thus only displaying the average shape. Whereas the overall shape is perfectly reproducible, the dark (i.e., dense) filament shows some variability. When extracting single exposures around 320 *μs* one can again find the cone-shaped envelope, but a variable dark pattern in the inside, see Fig. 3. It appears for most of the individual images and emanates from the laser focus point and seems to branch away from the target. In contrast to the rim of the first bubble, which also appears dark, this cannot be related to differential phase contrast as there are no corresponding bright structures. The dark lines are more likely to be caused by increased absorption, which, in turn, is caused by higher material density of the ejected material. It is hypothesised that after the collapse of the second bubble an inward motion of the water along the target surface takes place. This motion, together with a surface that is still hot dephases the movement of the upper and lateral part of the bubble, such that a jet-like flow transports material away from the substrate.

Such behavior is discussed by Philipp and Lauterborn^43^ who briefly mention a possible outward flow (possibly toroidally shaped) after bubble collapse. Their shadowgraphs also show signal of outward moving structures (microbubbles). As opposed to regular cavitation^43^ in our case the formation of an inward jet within the first bubble is not observed and, given the regime of the wetting angle being larger than 90 degrees^45^ is unlikely. This is coupled to bubble flattening rather than formation of an inward jet. A plume emission was reported by Tsuji *et al.*^18^, which takes place after collapse of the first bubble. Data after the second bubble was not reported, but similar hydrodynamic flow could govern both collapses. Concluding, we observe a thin filament or jet of outward moving material in a third bubble after the second bubble has collapsed. This pattern possesses considerable attenuation coefficient. Therefore massive emission of material is very likely. A sketch of this phenomenon is added to Fig. 3.

### Nanoparticle detection

**Figure 4:**
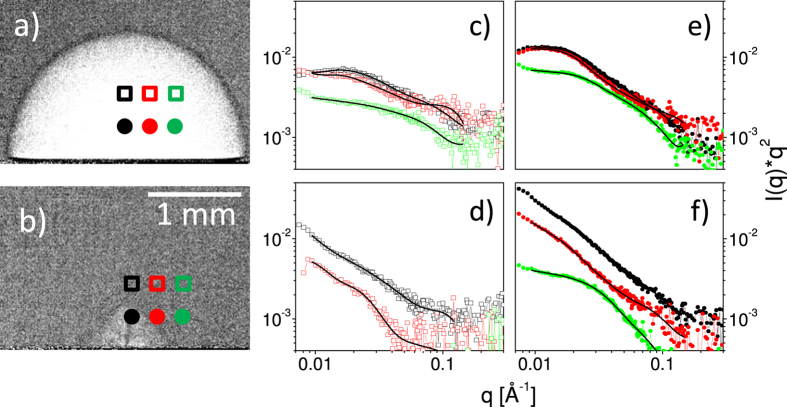
Selected radiographs at 110 *μs* (a) and 320 *μs* (b) with indicated locations for the SAXS measurements. Corresponding SAXS (**c**–**f**) curves within the Kratky representation (I(q) * q^2^) at the chosen points in time and space relative to the bubble formation are shown to the right of the two XR delays. The data at 0.8 mm height, (**c**,**d**), is drawn by open symbols, while data at 0.5 mm height, (**e**,**f**), is represented by full symbols. The topmost curves (highest intensity) are always at the center, followed by an increasing lateral displacement. The lines are fits to the data.

The examination of the mass content can be done by SAXS, which is sensitive to mass distributions with particle diameters in the range between a few up to hundreds of nanometers, depending on the resolution and scattering yield^17^,^24^. We have performed time-resolved SAXS with a time resolution sufficient for distinguishing between the different phases of the bubble motion. The abundance of primary and secondary particles, respectively as function of delay is shown in Fig. 2(c) together with the bubble shape^24^. In particular the first bubble and the jet phase were analysed in the SAXS experiment as a function of both height above the substrate and lateral displacement from the bubble center of rotation, see Fig. 4.

The resulting curves are fitted by the unified fit approach with free parameters being the particle size, polydispersity and fractal exponent describing the smoothness of the particles of each hierarchy level^49^. Additionally a scaling parameter matches the relative signal strength. We used two hierarchy levels in the order of 12 nm and 60 nm diameter, respectively. These have been identified earlier as primary, compact particles, and as larger, secondary particles^17^,^22^, respectively. The observed variability of secondary particle size points towards loose, reversible agglomeration^24^. Inspection by *post-mortem* electron microscopy also reveals the presence of large compact particles, that may contribute to the signal, but may have different origin.

The mass abundance is checked in a model-independent way by the Porod invariant. Figure 4(c–f) shows SAXS curves at the selected positions relative to the bubbles in radiographs (a) and (b). The first observation is that during development of the first bubble the weight of the scattering yield is located at higher q, see Fig. 4(c,e). This agrees with the higher ratio of primary versus secondary particles within the first bubble as reported before^24^. At larger delay the weight shifts towards lower q and larger agglomerates. More importantly, the scattering signal far from the center at 110 *μs* depends only weakly on the lateral position while it drops very quickly at 320 *μs*. Specifically at 0.8 mm from the center particle-related intensity is missing at 0.8 mm height and reduced by an order of magnitude at 0.5 mm height (Fig. 4(d,f)).

**Figure 5:**
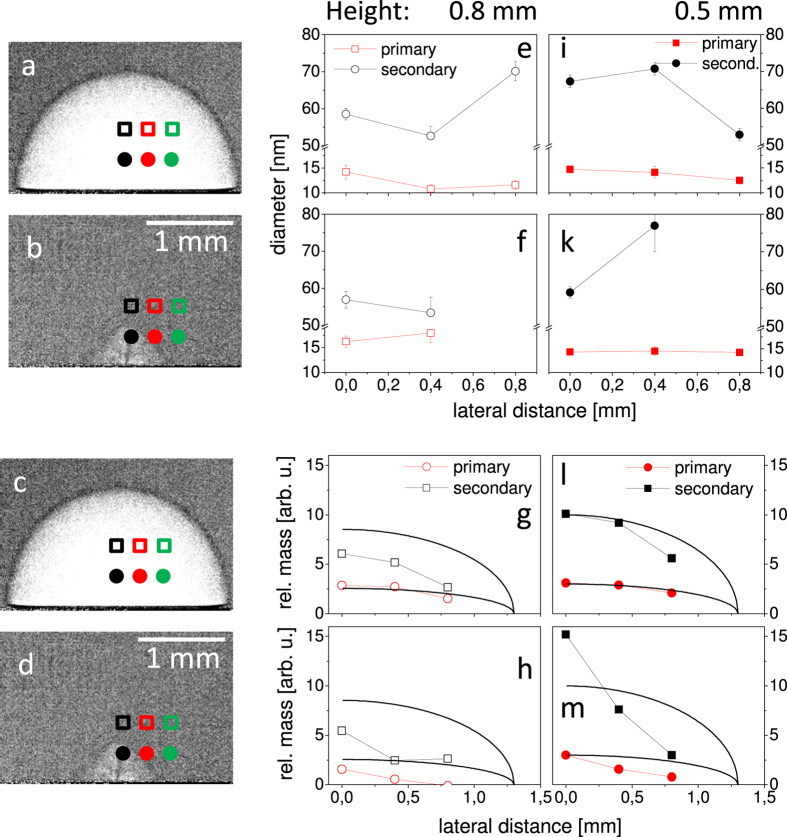
Selected radiographs at 110 *μs* (a,c) and 320 *μs* (b,d) with indicated locations of the SAXS analysis. Corresponding sizes of the fractions of primary and secondary particles at different positions within the bubble and the two time delays, (**e**,**f**,i,**k**), and the relative mass of these fractions, (**g**,**h**,**l**,**m**), as determined from the SAXS invariant at different positions within the bubble and two time delays. The full lines in (**g**,**h**,**l**,**m**) scale with the bubble thickness as guide to the eye at the respective height.

The Porod invariant is plotted for all measured positions as function of distance from the center in Fig. 5(g,h,l,m). Assuming a homogeneously filled bubble one would expect an invariant that changes with the thickness of the probed part of the bubble. Based on a spherical bubble cross section parallel to the target we added a guide to the eye for the intersected bubble thickness. Indeed within the first bubble the abundance of both primary particles and agglomerates/larger particles follow this line. This supports the notion that most of the mass is contained in the first bubble and distributed rather homogeneously (except for a slight agglomerate enrichment towards the center of the bubble). This is no longer true after bubble collapse. In the third bubble the mass is strongly concentrated at the center with a distribution width around 0.4 mm. The observed filament of ejected mass in the XR images is even more confined, but as the ejection procedure has some stochastic contribution averaging over a large number of shots possibly widens the distribution. We conclude that the filaments observed in the images correlate to ejection of a large amount of nanoparticulate mass along the rotation axis. This mass is strongly concentrated and contains large particles as it probably stems from the redeposited materials during bubble collapse.

The average sizes of the mass fractions do not vary strongly as a function of position, as observed earlier with rather stationary distributions. A slight growth of the primary particles for the later delay might be apparent, see Fig. 5(e,f,i,k). There is also a trend towards reduced sizes at lateral displacement (i. e. 15 nm towards 13 nm for primary particles). This adds to a picture that crystalline primary particles are formed very early in the ablation process, probably during the first bubble expansion, while further growth is only achieved through remaining atomic, ionic or cluster species below the current detection limit for smallest particles. The major part of kinetics of the condensed mass during bubble dynamics is dominated by the interaction of primary particles to form secondary larger particles. The different trajectories of each fraction during redeposition and final suspension in the medium may then further influence the final particle size and their surface chemistry.

## Summary

The optimisation of PLAL towards size^3^ and yield control^50^ of the harvested nanoparticles demands an understanding of the primary processes. This can only be achieved by a simultaneous observation of macroscopic and nanoscopic kinetics. The interplay between redeposition and release in the liquid is still puzzling. This study has combined time-resolved X-ray methods to study the structural kinetics of particle formation temporally and spatially correlated to the macroscopic bubble formation process during PLAL.

The X-ray radiography delivers a video sequence of the whole cavitation dynamics including insight into the interior of the bubble. Particle growth dynamics and spatial distribution have been quantified based on X-ray small angle scattering, with its signal intensity proportional to particle mass or volume rather than particle number. It is confirmed that within the first bubble primary particles of (mass-weighted) diameters in the range of 12–15 nm are predominant and fill the bubble volume homogeneously.

After partial redeposition of the trapped matter a strong growth and agglomeration of the nanoparticles occurs. This agglomeration can be partly reversible, but can also favor sintering on a later stage for irreversible size increase. The second bubble shows inhomogeneous contrast features, bearing a hemispheric volume of lower density inside. Condensed mass is ejected in particular via a directed jet forming from the target in a third bubble, which is triggered by the particular shape and water flow of the vapor bubble during collapse. Unlike cavitation processes of gas bubbles near a wall as reported in literature no inward jet is formed during collapse. The dynamics reflect a wetting angle at the bubble liquid-solid line, which is larger than 90 degrees. This leads to a flatting of the collapsing bubble. Consequently, material redeposits already in the first bubble. Subsequently a lateral drag develops, which finally leads to a jet emission away from the target to release the ablated solid material into the liquid. This jet is imaged inside the irregularly shaped third bubble where it extends from the target to the bubble tip with a vertical length of about a half millimetre. A contribution to the large contact angle may be given by the still heated target, which hinders liquid wetting and disturbs the radially symmetric bubble motion.

With this scenario we find a comprehensive description of mass transport during PLAL. The processes of agglomeration and growth versus small primary particle release are decisive for the morphology and quality of the harvested colloidal particles. Therefore one parameter in size tailoring may be the modification of the bubble dynamics. PLAL from wire targets has been reported together with the associated bubble dynamics^14^. The strong change of drag geometry hinders the efficient redeposition, which, in turn, significantly increases ablation efficiency and yield. Furthermore, the pathway of size-stabilizing ligands from the liquid can be rationalized. The present study could pave the way towards better correlation of hierarchical processes of the interaction of the macroscopic bubble formation and nanoparticle appearance, growth and release.

## Methods

### Materials

Ultrapure water (MilliQ, Millipore) was circulated through a membrane pump with pressure buffer vessel within the chamber. Pure silver ribbon (99.9%) was moved continuously for the SAXS experiment, while a fixed silver plate (99.99%) was irradiated for the XR with less than 100 shots before changing the target.

**SAXS** was performed at the cSAXS beamline at the Swiss Light Source (PSI Villigen, CH) with a pencil beam of 6 × 24 *μm* cross section. A pixel X-ray detector (Pilatus 2 M^51^) was used. It could be gated active with square-well pulses to limit the recording to short intervals in time with fixed delay to the laser impact. A gate width of 60 *μs* was used while the delay could be varied at will. The counts were accumulated for a large number 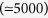 of shots for a single two-dimensional SAXS image. Images with positive delays (i.e., when the laser arrives prior to the X-rays) were compared to images with negative delay to remove static background signal, e. g. from pre-existing nanoparticles. Analysis is done for one-dimensional scattering curves I(q) with 
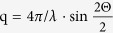
 at an X-ray wavelength of *λ* = 1.107 Å as function of scattering angle 2Θ. Particle size fitting was performed by a generalized algorithm^49^, without explicit input of particle shape and inclusion of particle aggregation. The Porod invariant is the integral over the weighted scattering intensity I(q) * q^2^ and proportional to the total scattering mass in the limit of diluted particles^52^,^53^.

**Fast X-ray radiography** was performed at the beamline TOPO-TOMO at the ANKA synchrotron (KIT Karlsruhe, D). A broad beam directly from the bending magnet source was spectrally filtered by a silicon wafer and its size was set by slits to 4 × 4 mm. The white beam was used only with further filtering by the beamline beryllium window (0.25 mm) and the X-ray windows and the water column inside the chamber. The median energy was estimated to be 12 keV. The X-rays transmitted through the chamber were detected by a scintillator (LuAG:Ce) and recorded with a lens-coupled camera system (PCO.dimax). The active-pixel camera allowed for a high frame rate, depending on the crop size. We used a crop of 432 × 248 pixels for a 28.5 kHz frame rate, 20 *μs* exposure time and an effective pixel size due to the 1.5× magnification optics of 7.3 *μm*. Due to the reproducibility of the process the recordings from typically 24 laser shots could be added up. Several films with short exposures times could be interleaved *post-mortem* to achieve an effective frame rate of 85 kHz. Standard image correction such as dark count subtraction and flat-field correction were done with the software ImageJ^54^.

**Ablation** was performed in a flow chamber as described previously^24^. A liquid volume was formed by a stainless steel body with two polyimide windows (Kapton, DuPont) pressed against it from the sides. The laser beam entered the volume through a plano-convex lens acting as vessel wall. Water was continuously pumped to ensure an exchange of the liquid in the region of interest (maximum bubble extension) between two laser shots at either 100 Hz (SAXS) or single shots (XR). Thereby secondary radiative particle modification can be avoided^55^,^56^. The silver target was transported continuously through the laser focus in the first case, while in the second case a fixed (fresh) target was used every 100 shots. This arrangement was useful in order both to remove permanent gas bubbles from the laser path and assure a homogeneous flow of liquid.

The excitation laser for SAXS was a Innolas Spitlight DPSS-250-100 nanosecond laser with 10 mJ pulse and 0.1 mm focus diameter (1.2 kJ/m^2^) at 100 Hz repetition rate. For the X-ray radiography a Continuum Nd:YAG laser with up to 40 mJ and 10 Hz maximum repetition rate was employed. Pulse energy was set to 20 mJ, with a fluence of 640 J/m^2^ at 0.2 mm focus diameter.

### Calculations

The bubble dynamics are compared to the solution of the Rayleigh-Plesset equation^37^ with inclusion of water viscosity and surface tension, by converting into a first-order differential equation, which is solved with appropriate boundary conditions^41^,^57^. The solutions are not unique, but a good fit of the first-bubble motion is achieved with polytropic exponent of 1.15, smallest and largest bubble size of 0.09 mm and 1.2 mm, respectively. The initial conditions were starting bubble size of 0.24 mm and starting velocity of 12 m/sec. No combination of parameters allowed for the fitting of the second, shorter bubble lifetime or the third oscillation.

## Additional Information

**How to cite this article**: Ibrahimkutty, S. *et al.* A hierarchical view on material formation during pulsed-laser synthesis of nanoparticles in liquid. *Sci. Rep.* **5**, 16313; doi: 10.1038/srep16313 (2015).

## Supplementary Material

Supplementary Video

Supplementary Information

## Figures and Tables

**Figure 1:**
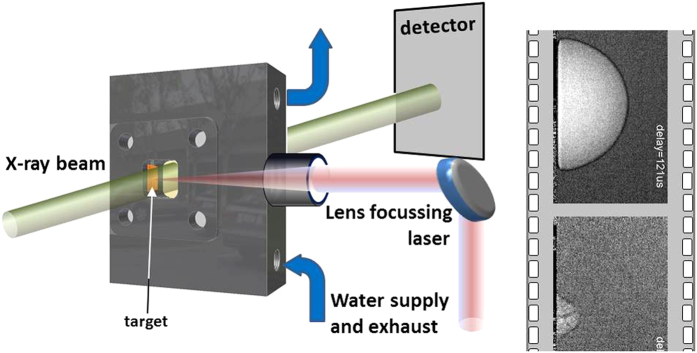
Radiographic and scanning SAXS tracking of material distribution during the ablation procedure during pulsed-laser assisted nanoparticle synthesis in liquids. Either a monochromatic pencil beam is used for scanning SAXS or a wide white X-ray beam for radiography.
