# Supplementary material for: A hierarchical view on material formation during pulsed-laser synthesis of nanoparticles in liquid
Source: Sci Rep. 2015 Nov 9;5:16313. doi: 10.1038/srep16313 (PMC4637878; doi:10.1038/srep16313)
Supplement: Supplementary Information [file srep16313-s2.pdf]

Supplementary information for

"A hierarchical view on material formation during pulsed-laser synthesis of nanoparticles in liquid"

by

Shyjumon Ibrahimkutty[1,2], Philipp Wagener[3], Tomy dos Santos Rolo[1], Dmitry Karpov[1,4],Andreas Menzel[5], Tilo Baumbach[1,6], Stephan Barcikowski[3], and Anton Plech[1],

[1]ANKA/Institute for Photon Science and Synchrotron Radiation, KIT Karlsruhe, Germany

[2]current affiliation: MPI for Solid State Science, Stuttgart, Germany

[3]CENIDE and Dept. of Chemistry, University Duisburg-Essen, Germany

[4]current affiliation: Tomsk Polytechnic University, 30 Lenin Avenue, 634050 Tomsk, Russian Federation

[5]Swiss Light Source, Paul-Scherrer Institute Villigen, Switzerland

[6]Laboratory for Application of Synchrotron Radiation, KIT Karlsruhe, Germany

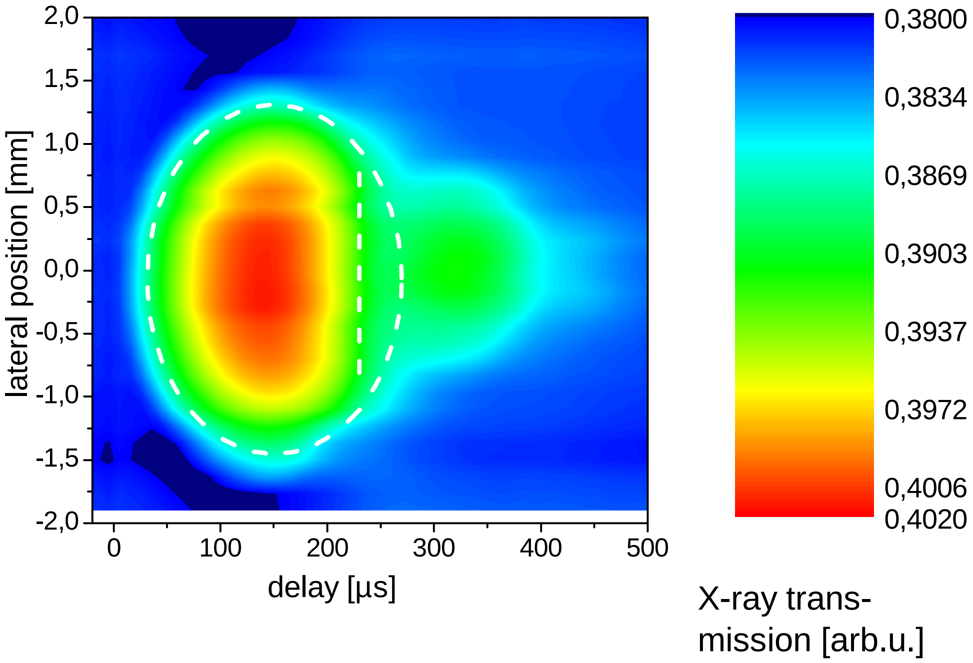

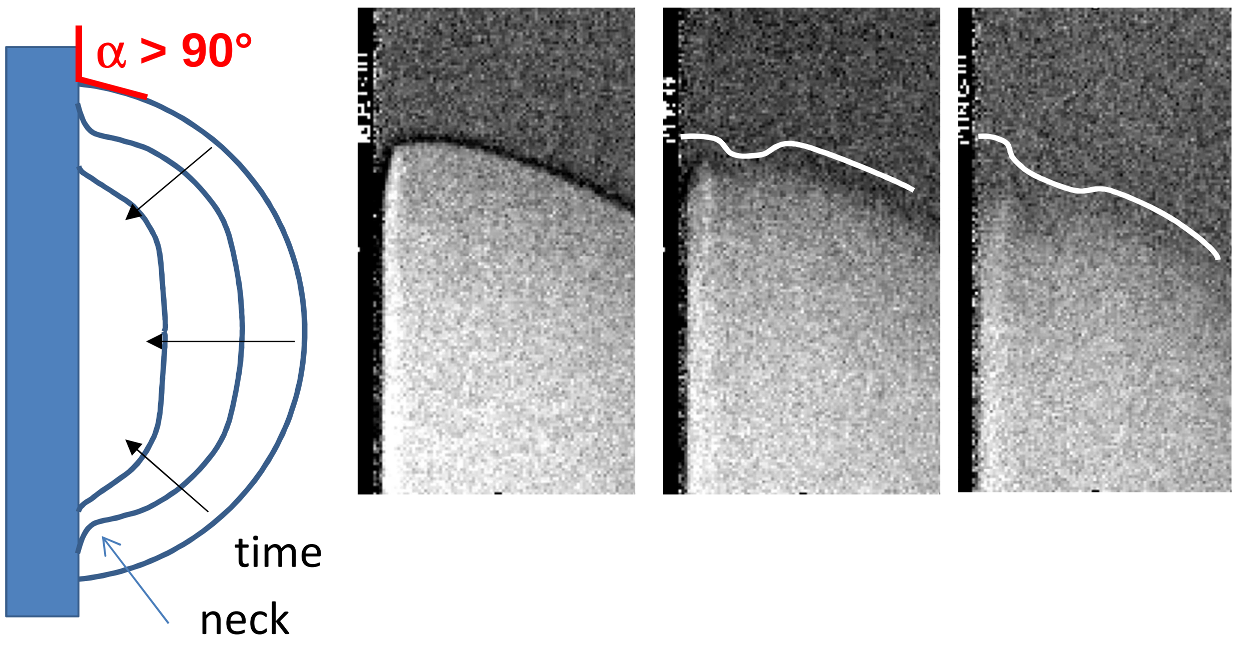

Supplementary figure S1: Upper: False color plot of the x-ray transmission with a scanned pencil beam during SAXS acquisition at fixed height above the target of 0.5 mm. A symmetric growth and collapse of the first bubble would be marked by an ellipsoidal boundary of abruptly changing transmission, as indicated by the dashed ellipse. During the collapse phase at a delay of about 200-260 µs the retracting bubble disappears at all lateral positions at equal delay (vertical line as guide for the eye). This means that the bubble flattens during collapse.

Figure S1: Lower: Sketch of the collapse of the first bubble according to Shima and Sato, Ingenieur Archiv 48 (1979) 85, with bubble surfaces at different points in time. Crops of the X-radiography film at 110, 133 and 156 µs are shown on the right together with an indication for the neck formation.

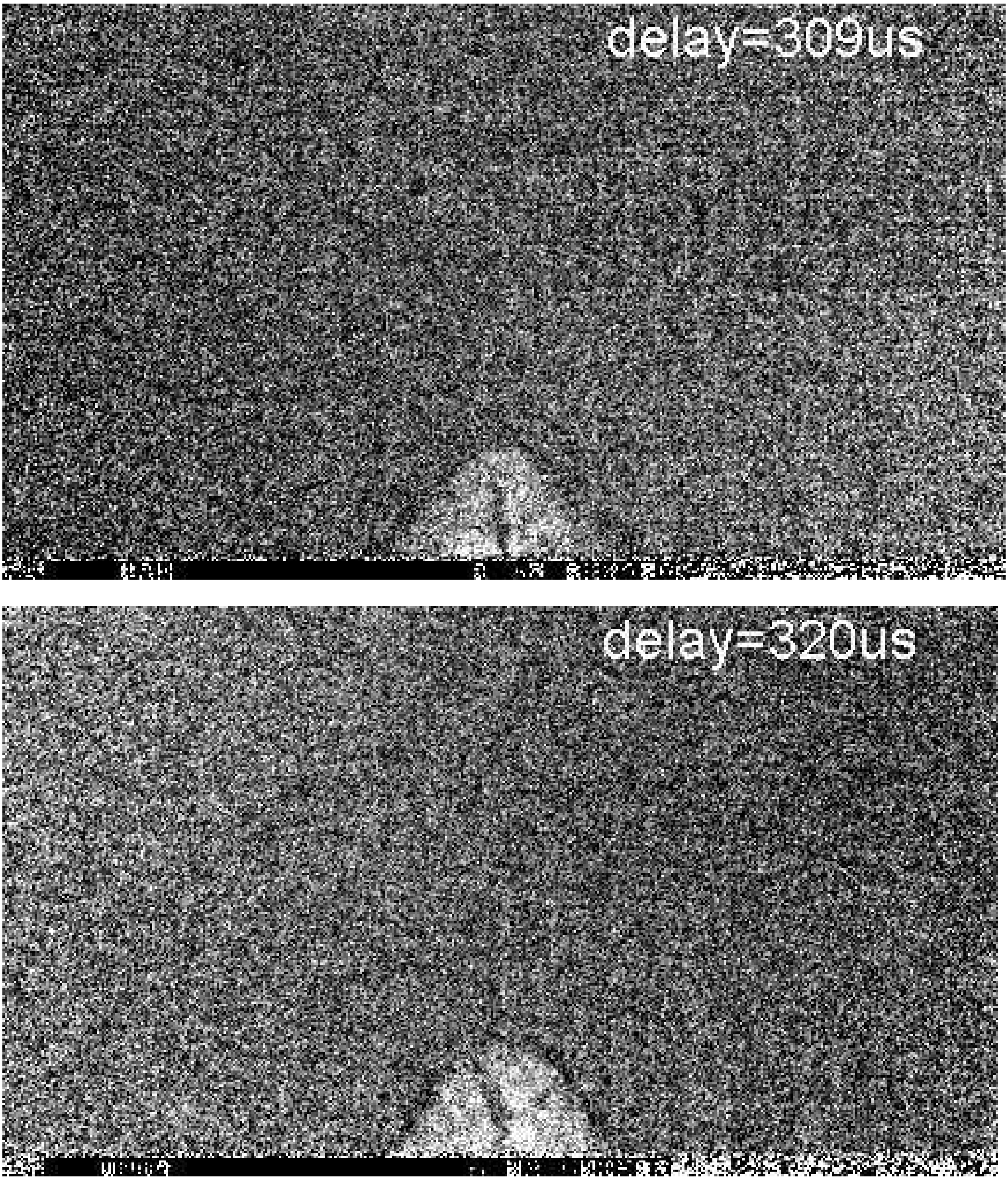

Supplementary figure S2: Contrast-enhanced images from figure 2, delay at 309 µs and 320 µs.

Supplementary video: Radiography sequence with 88 000 frames per second during the ablation process. The delay of the exposure relative to the laser impact on the target is displayed as inset. The target is positioned horizontally at the lower edge of the frames. The laser arrives centrally from the top.
